# Ophthalmic Sequelae of Ebola Virus Disease in Survivors, Sierra Leone

**DOI:** 10.3201/eid3012.240425

**Published:** 2024-12

**Authors:** Charlene H. Choo, Laura Ward, Ian Crozier, Tolulope Fashina, Daisy Yan, Brent R. Hayek, Caleb Hartley, Matthew Vandy, John G. Mattia, Lloyd Harrison-Williams, Jalikatu Mustapha, Carolyn Drews-Botsch, Steven Yeh, Jessica Shantha

**Affiliations:** University of Nebraska Medical Center, Omaha, Nebraska, USA (C.H. Choo, T. Fashina, C. Hartley, S. Yeh); Emory University, Atlanta, Georgia, USA (L. Ward, S. Yeh, J. Shantha); Frederick National Laboratory for Cancer Research, Frederick, Maryland, USA (I. Crozier); University of California, San Francisco, California, USA (D. Yan, J. Shantha); North Georgia Eye Clinic, Gainesville, Georgia, USA (B.R. Hayek); Ministry of Health and Sanitation, Freetown, Sierra Leone (M. Vandy, J.G. Mattia, L. Harrison-Williams, J. Mustapha); George Mason University, Fairfax, Virginia, USA (C. Drews-Botsch)

**Keywords:** Ebola virus disease, Ebola virus, Zaire ebolavirus, uveitis, eye health, ophthalmology, vision, Sierra Leone, viruses

## Abstract

The Ebola virus disease (EVD) outbreak of 2013–2016 was large, leaving in its wake an estimated 17,000 survivors in West Africa. Uveitis is one of the most common ophthalmic manifestations of EVD, but long-term follow-up in the at-risk population is lacking. We conducted a retrospective cross-sectional study of 521 EVD survivors from Sierra Leone who underwent comprehensive ophthalmic examination a median of 1,289 days, or ≈3.5 years, after discharge from Ebola treatment units. The most common ophthalmic findings were cataracts (117 eyes, 11.2%), uveitis (86 eyes, 8.3%), dry eyes (81 eyes, 7.8%), and chorioretinal scar (68 eyes, 6.5%). EVD survivors with cataracts, uveitis, optic neuropathy, and corneal scar were more likely to have vision impairment, defined as Snellen visual acuity worse than 20/50. Results of our study highlight the need for ongoing vision care in EVD survivors.

The Ebola virus disease (EVD) outbreak in West Africa during 2013–2016 was large, resulting in 28,652 cases, 11,325 deaths, and ≈17,000 survivors, primarily from Guinea, Liberia, and Sierra Leone (*1*). Although the epidemic formally ended in 2016, EVD poses an ongoing threat to public health. Another large EVD outbreak occurred in 2018–2019, causing 3,470 cases and 2,287 deaths in Democratic Republic of the Congo (DRC) (*1*,*2*), and another in 2022 led to 164 cases and 55 deaths in Uganda (*1*,*3*). All EVD outbreaks in West Africa, except for the 1994 outbreak of Taï Forest ebolavirus, have been caused by *Zaire ebolavirus*, the *Ebolavirus* species with the highest fatality rate (40%–90%) (*1*,*4*). During a *Zaire ebolavirus* outbreak in DRC in 1995, bilateral conjunctival injection was reported in 48% of hospitalized patients; late ocular findings including uveitis were described in a few surviving patients (*5*,*6*). However, the ophthalmic manifestations of EVD were poorly understood before the West Africa EVD outbreak.

Studies on the large number of EVD survivors from the epidemic in 2013 revealed uveitis as the most common ophthalmic sequela. In Liberia, where EVD caused 10,678 cases and 4,810 deaths, uveitis was found in 26% of survivors ≈1 year after symptom onset and in 33% at the 2-year follow-up (*7*). Another study found that ≈40% of the EVD survivors with uveitis were blind, with visual acuity (VA) of 20/400 or worse (*8*). In Guinea, where EVD caused 3,814 cases and 2,544 deaths, ocular complications were reported in 18% of survivors and uveitis in 13.5% (*9*,*10*). Within Sierra Leone, uveitis was reported in 18%–34% of survivors and was associated with worse VA and higher viral load when they sought care for acute systemic presentation (*11*,*12*). Pediatric EVD survivors also experienced uveitis more frequently than close contact controls (10.8% vs. 1.7%; p = 0.03) and had worse vision-related quality of life (*13*).

In this study, we report the prevalence of long-term ophthalmic findings in the largest long-term cohort of EVD survivors from Sierra Leone who underwent comprehensive ophthalmic examination ≈3.5 years after resolution of their acute illness. We also describe vision impairment associated with uveitis, in addition to the range of ophthalmic manifestations we observed.

## Methods

### Patient Recruitment and Evaluation

We conducted this study in partnership with the Ministry of Health and Sanitation, Sierra Leone; Emory University (Atlanta, GA, USA); and nongovernmental organizations including Partners in Health, John Snow, Inc., and Central Global Vision Fund. We obtained Institutional Review Board approval for review of retrospective data from Emory University and the Office of Ethics and Scientific Review Committee, Sierra Leone Ministry of Health and Sanitation. We conducted human subject research in accordance with the tenets of the Declaration of Helsinki.

EVD survivors were assessed for ophthalmic complications at the Lowell and Ruth Gess Kissy Eye Hospital (Freetown, Sierra Leone) in June 2018. Patients from outlying districts were examined at the Makeni Government Hospital Eye Clinic (Makeni, Sierra Leone). Full ophthalmic exams were performed and included corrected VA, slit lamp, and dilated funduscopic examination. B-scan ultrasound was performed when clinically indicated due to media opacity (i.e., cataract, vitreous opacity) that precluded a view of the posterior fundus. We confirmed EVD survivor status with survivor certificates or a history of EVD requiring Ebola treatment unit (ETU) admission. We collected demographic information, past medical and ocular history, and current systemic and ocular symptoms during a comprehensive medical interview. We defined active uveitis as the presence of inflammation in the anterior chamber, keratic precipitates, vitreous haze, or retinal or choroidal infiltrates. We graded uveitis according to the Standardization of Uveitis Nomenclature and National Eye Institute guidelines for anterior chamber cell, flare, vitreous cell, and haze (*14*,*15*). Data reviewed included past medical and ocular history, ETU admission and discharge dates, and ocular and systemic symptoms during acute EVD.

### Statistical Analysis

We analyzed demographic and medical history by patient; we summarized ophthalmic data on a per-patient and per-eye basis. For ophthalmic data summarized per-person, if the condition was found in either eye, we counted the patient as having the finding. We summarized descriptive statistics as frequencies for categorical data and mean/SD or median/interquartile range (IQR) for continuous data. We conducted bivariate analysis of factors associated with uveitis using χ^2^ test for per-person analysis and unadjusted generalized estimating equations, controlling for the correlation between eyes, for per-eye analysis.

We defined vision impairment as Snellen VA of 20/50 or worse. We converted Snellen VAs to logarithm of the minimum angle of resolution (logMAR) values for all analyses. We calculated mean logMAR VA by eye, as well as by the better-seeing eye and worse-seeing eye. We excluded eyes with no light perception vision (n = 7) from analysis because there is no appropriate logMAR conversion; however, we recognize this exclusion could bias the analyses toward accepting the null hypothesis (*16*). We created a multivariable model to examine the relationship between vision impairment status and ophthalmic findings of interest and adjusted for cataract, uveitis, corneal scar, and optic neuropathy. We adjusted the correlation between eyes using a compound symmetric correlation structure. We performed all analyses using SAS version 9.4 (SAS Institute Inc., https://www.sas.com); we considered α<0.05 statistically significant.

## Results

### Demographic and Clinical Characteristics

We included 521 EVD survivors from Sierra Leone in this study (Table 1). The cohort had a mean age of 31.1 years (SD 16.0 years); 285 (54.7%) patients were female and 236 (45.3%) male, predominantly of Temne ethnicity (61.2%). Most EVD survivors who were offered eye care services were recruited from the Western Area urban or rural districts (58.4%) or the Northern Province (29.0%).

**Table 1:** Demographic characteristics of survivors in study of ophthalmic sequelae after Ebola virus disease, Sierra Leone*

Characteristic	Total, N = 521
Age, mean (SD)	31.1 (16.0)
Sex, no.	
F	285 (54.7)
M	236 (45.3)
Ethnicity	n = 485
Temne	297 (61.2)
Limba	64 (13.2)
Mende	44 (9.1)
Fula	18 (3.7)
Other	62 (12.8)
Education level	n = 470
Primary and junior secondary school	177 (37.7)
None or nursery school	175 (37.2)
Higher education	118 (25.1)
Occupation	n = 492
Farmer or trader	213 (43.3)
Student	143 (29.1)
Unemployed or housewife	27 (5.5)
Healthcare or social worker	24 (4.9)
Construction worker	18 (3.7)
Other	67 (13.6)
District of residence	n = 510
Western Area Urban	157 (30.8)
Western Area Rural	141 (27.6)
Bombali or Tonkolili, Northern Province	148 (29.0)
Koidu or Kono, Eastern Province	52 (10.2)
Other	12 (2.4)

*Values are no. (%) except as indicated. ETU, Ebola treatment unit,

### Patient Medical History and Current Symptoms

The cohort had a median duration of stay in an ETU of 22 (IQR 14–30) days (Table 2). They were examined after a median of 1,289 days (IQR 1,207–1,371), or ≈3.5 years, after ETU discharge. Many survivors reported a history of malaria (47.8%) or typhoid (92.7%) during their lifetime. Approximately 87% of the cohort had >1 previous eye exam. Fifty-one survivors (10.0%) reported a previous diagnosis of uveitis and 42 survivors (8.2%) a previous diagnosis of cataract.

**Table 2:** Medical history and current symptoms of survivors in study of ophthalmic sequelae after Ebola virus disease, Sierra Leone*

Medical history and symptoms	Total, n = 521
Days admitted in ETU, median (IQR)	22 (14–30)
Days from ETU discharge to study enrollment, median (IQR)	1,289 (82)
Had previous eye exam	444 (86.6)
Medical history	n = 510
Malaria	244 (47.8)
Typhoid	473 (92.7)
Lassa fever	7 (1.4)
HIV	3 (0.6)
Other	54 (10.6)
None	10 (2.0)
Ocular history	n = 510
Uveitis	51 (10.0)
Cataract	42 (8.2)
Current systemic symptoms	n = 510
Any systemic symptoms	503 (98.6)
Headache	485 (95.1)
Joint pain	427 (83.7)
Fatigue	369 (72.4)
Chest pain	316 (62.0)
Low mood	305 (59.8)
Abdominal pain	294 (57.6)
Weight loss	276 (54.1)
Joint stiffness and/or swelling	258 (50.6)
Anxiety	235 (46.1)
Current ocular symptoms	N = 510
Any eye symptoms	484 (94.9)
Eye pain	377 (73.9)
Light sensitivity	344 (67.4)
Blurred vision	342 (67.1)
Tearing	298 (58.4)
Eye redness	270 (52.9)
Floaters	152 (29.8)
Vision loss	134 (26.3)

*Values are no. (%) except as indicated. ETU, Ebola treatment unit; IQR, interquartile range.

Most EVD survivors (96.5%) had systemic symptoms, such as headache (95.1%), joint pain (83.7%), fatigue (72.4%), chest pain (62.0%), low mood (59.8%), abdominal pain (57.6%), weight loss (54.1%), joint stiffness/swelling (49.5%), and anxiety (46.1%). Most survivors (92.9%) also had ocular symptoms, such as eye pain (73.9%), light sensitivity (67.4%), blurred vision (67.0%), tearing (58.4%), eye redness (52.9%), floaters (29.8%), and vision loss (26.2%).

### Ophthalmic Examination and Diagnosis

**Figure 1:**
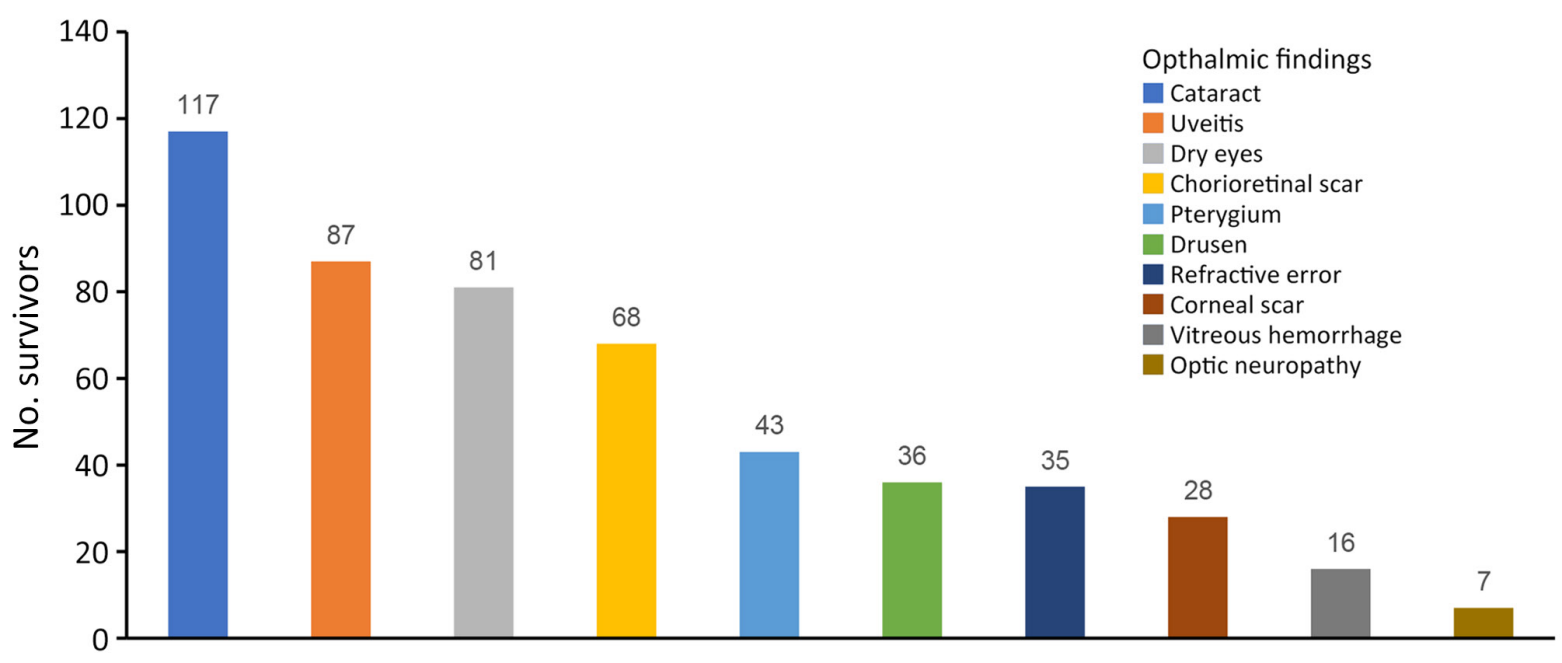
Common ophthalmic findings in Ebola virus disease survivors, Sierra Leone. The most common were cataract (11.2%), uveitis (8.3%), dry eyes (7.8%), chorioretinal scar (6.5%), and pterygium (4.1%).

**Figure 2:**
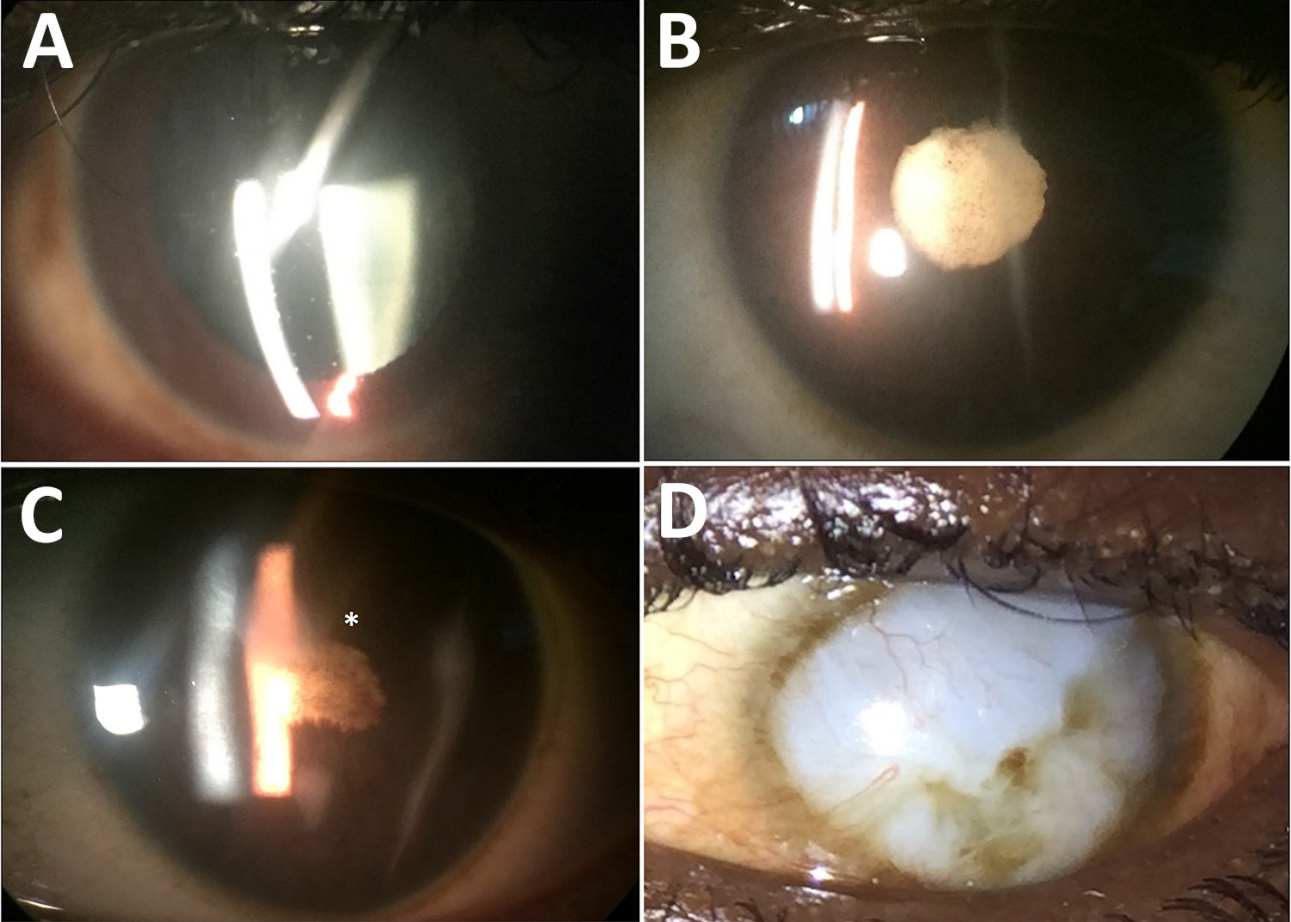
Anterior segment photographs showing the spectrum of ophthalmic sequelae associated with EVD in survivors, Sierra Leone. A) A patient with anterior uveitis has diffuse round keratic precipitates on the corneal endothelium, predominantly within the inferior cornea. B) An EVD survivor with severe, chronic uveitis has posterior synechiae, pigment on the lens capsule, and a dense cataract. C) Another EVD survivor with severe uveitis has dense posterior synechiae overlying a cataract, leading to blindness, and corneal edema involving the superior paracentral cornea (asterisk). D) An external photograph shows a diffuse corneal opacity (leukoma) with superior neovascularization, which was not present before the onset of acute EVD. EVD, Ebola virus disease.

**Figure 3:**
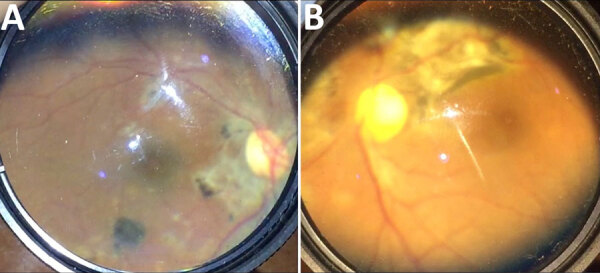
Posterior and fundus photographs showing the spectrum of ophthalmic sequelae associated with EVD in survivors, Sierra Leone. A) Posterior segment photograph of an EVD survivor shows peripapillary chorioretinal scarring and variable pigmentary changes indicative of multifocal choroiditis. B) Fundus photograph of another survivor shows dense chorioretinal scarring along the superotemporal arcade and nasal to the nerve, consistent with inactive posterior uveitis. EVD, Ebola virus disease.

A total of 358 eyes (34.4%) had ophthalmic findings (Table 3; Figure 1). The most common diagnoses were cataract (117 [11.2%]), uveitis (86 [8.3%]), dry eye (81 [7.8%]), chorioretinal scar (68 [6.5%]), and pterygium (43 [4.1%]) (Figures 2, 3). Anterior uveitis was the most common location (44 [51.2%]), followed by panuveitis (17 [19.7%]) and posterior uveitis (16 [18.6%]).

**Table 3:** Ophthalmic findings in survivors in study of ophthalmic sequelae after Ebola virus disease, Sierra Leone

Ophthalmic findings	No. (%) patients, n = 521	No. (%) eyes, n = 1,042
Any ophthalmic finding	200 (38.4)	358 (34.4)
Cataract	62 (11.9)	117 (11.2)
Uveitic cataract	10 (16.1)	17 (14.5)
Uveitis	69 (13.2)	86 (8.3)
Anterior uveitis	34 (49.3)	44 (51.2)
Intermediate uveitis	1 (1.4)	1 (1.2)
Anterior/intermediate uveitis	4 (5.8)	5 (5.8)
Posterior uveitis	13 (18.8)	16 (18.6)
Panuveitis	15 (21.7)	17 (19.8)
Unspecified	2 (2.9)	3 (3.5)
Dry eyes	41 (7.9)	81 (7.8)
Chorioretinal scar	56 (10.7)	68 (6.5)
Pterygium	20 (3.8)	43 (4.1)
Drusen	20 (3.8)	36 (3.5)
Refractive error	18 (3.5)	35 (3.4)
Corneal scar	22 (4.2)	28 (2.7)
Vitreous hemorrhage	9 (1.7)	16 (1.6)
Optic neuropathy	6 (1.2)	7 (0.7)

### Univariable Analysis in Patients with or without Uveitis

We analyzed demographic and clinical characteristics of the cohort by patients’ uveitis status (Tables 4, 5). EVD survivors who were female (p = 0.036) and older (p = 0.048) with a previous history of eye exam (p = 0.006), previous diagnosis of cataracts (p<0.001), previous diagnosis of uveitis (p = 0.030), and symptoms of vision loss (p = 0.026) were more likely to have uveitis. The presence of uveitis was not associated with days spent in the ETU (p = 0.182). Survivors with bullous keratopathy (p<0.001), chorioretinal scar (p = 0.012), posterior synechiae (p = 0.002), retinal detachment (p = 0.012), and vitreous opacity (p = 0.022) were significantly more likely to have uveitis. Cataracts (p = 0.065), corneal scar (p = 0.276), and optic neuropathy (p = 0.790) were not significantly associated with the presence of uveitis. Finally, survivors with vision impairment, defined as Snellen VA worse than 20/50, were significantly more likely to have uveitis (p = 0.004).

**Table 4:** Demographic and clinical characteristics associated with uveitis status in survivors in study of ophthalmic sequelae after Ebola virus disease, Sierra Leone*

Characteristics	Total, N = 521	Uveitis, n = 69	No uveitis, n = 452	OR (95% CI)	p value
Age, mean (SD), n = 496	31.1 (16.0)	29.7 (12.7)	31.3 (16.5)	0.97 (0.95–0.99)	0.048
Sex, N = 510				1.78 (1.03–3.05)†	0.036
F	285 (55.9)	46 (16.1)	239 (83.9)		
M	236 (45.3)	13 (9.7)	223 (90.3)		
Days in ETU, mean (SD), n = 184	26.8 (21.4)	22.7 (12.3)	27.4 (22.5)	0.99 (0.97–1.01)	0.182
Previous eye exam, n = 513	444 (86.5)	67 (15.1)	377 (84.9)	5.95 (1.42–24.88)	0.006
History of cataract, n = 510	42 (8.2)	16 (38.1)	26 (61.9)	4.82 (2.43– 9.56)	<0.001
History of uveitis, n = 510	51 (10.0)	12 (17.4)	39 (8.8)	2.17 (1.07–4.39)	0.030
Vision loss symptoms, n = 510	134 (26.3)	26 (19.4)	108 (21.2)	1.86 (1.09–3.18)	0.026

*Values are no. (%) except as indicated. ETU, Ebola treatment unit; OR, odds ratio. †OR is the odds of uveitis for women vs. men.

**Table 5:** Ophthalmic examination findings associated with uveitis status in eyes of survivors in study of ophthalmic sequelae after Ebola virus disease, Sierra Leone*

Ophthalmic exam finding	No. (%) eyes			OR (95% CI)	p value
	Total, N = 1,042	Uveitis, n = 86	No uveitis, n = 956		
Bullous keratopathy	2 (0.2)	1 (50.0)	1 (50.0)	11.13 (8.67–14.51)	<0.001
Cataract	117 (11.2)	14 (12.0)	103 (88.0)	1.94 (0.96–3.89)	0.065
Corneal scar	28 (2.7)	5 (17.9)	23 (82.1)	1.94 (0.59–6.37)	0.276
Chorioretinal scar	68 (6.5)	17 (25.0)	51 (75.0)	3.74 (1.84–7.62)	0.012
Optic neuropathy	7 (0.7)	6 (85.6)	1 (14.3)	1.53 (0.07–34.47)	0.790
Posterior synechiae	7 (0.7)	3 (42.9)	4 (57.1)	11.58 (2.23–55.98)	0.002
Retinal detachment	7 (0.7)	3 (42.9)	4 (57.1)	5.64 (1.46–21.67)	0.012
Vitreous opacity	21 (2.0)	8 (38.1)	13 (61.9)	5.41 (2.04–14.45)	0.022
Visual acuity					
20/40 or better	783 (75.1)	54 (6.9)	729 (93.1)	2.25 (1.32–3.83)	0.004
20/50 or worse	259 (24.9)	32 (12.4)	227 (87.6)	NA	NA

*NA, not applicable; OR, odds ratio.

### Visual Acuity and Multivariable Analysis by Vision Impairment Status

A total of 259 (24.9%) of the 1,042 eyes examined had vision impairment, or Snellen VA worse than 20/50. Vision impairment was found in 18.0% of better-seeing eyes and 32.0% of worse-seeing eyes. The median logMAR VA was 0.23 (Snellen VA 20/34) in all eyes, 0.19 (Snellen VA 20/30) in the better-seeing eyes, and 0.30 (Snellen VA 20/40) in the worse-seeing eyes. Multivariable analysis revealed that the presence of vision impairment was significantly associated with cataract (odds ratio [OR] = 7.68; p<0.001), uveitis (OR = 2.08; p = 0.007), corneal scar (OR = 4.23; p = 0.001), and optic neuropathy (OR = 6.32; p = 0.034) (Table 6).

**Table 6:** Multivariable model of ophthalmic findings and vision impairment in eyes of survivors in study of ophthalmic sequelae after Ebola virus disease, Sierra Leone*

Ophthalmic finding	No. (%) eyes		OR (95% CI)	p value
	Vision impairment, n = 259	No vision impairment, n = 783		
Cataract	71 (27.4)	46 (5.9)	7.68 (4.32–13.64)	<0.001
Uveitis	32 (12.4)	54 (7.0)	2.08 (1.23–3.52)	0.0065
Corneal scar	12 (4.6)	16 (2.0)	4.23 (1.80–9.95)	0.0010
Optic neuropathy	5 (1.9)	2 (0.3)	6.32 (1.15–34.69)	0.0335

*Vision impairment was defined as visual acuity worse than 20/50 Snellen. OR, odds ratio.

## Discussion

This retrospective observational study included a large cohort of 521 EVD survivors who underwent comprehensive ophthalmic examination at long-term follow-up, ≈3.5 years after their initial ETU admission for acute EVD. Results of the study demonstrate a broad spectrum of ophthalmic findings that were associated with vision impairment, including cataract, uveitis, corneal scar, and optic neuropathy.

Uveitis remained one of the most common ophthalmic diagnoses found, affecting 8.4% of EVD survivors. The prevalence of uveitis in our study was lower than in other studies from Sierra Leone also conducted during the West Africa EVD outbreak, which reported uveitis in 18%–34% of EVD survivors a few months after ETU discharge (*8*,*11*,*12*). In those studies, EVD survivor status was determined on the basis of EVD survivor certificates, history of ETU admission, and laboratory diagnostics when available. Some EVD survivors in our cohort might have been treated for uveitis in the previous few years; 87% reported having >1 previous eye exam, which was also associated with the presence of uveitis (p = 0.05). Another explanation is that EVD-associated uveitis develops shortly after the acute illness; risk declines over time, potentially in relation to the viral load in ocular tissues. One previous study showed that a higher viral load at acute EVD illness was associated with the development of uveitis (*11*). Viable Ebola virus was detected from the ocular fluid of an EVD survivor at 14 weeks in association with panuveitis but was no longer detected at 27 months, when the uveitis had been inactive for 3 months (*17*). In the Ebola Virus Persistence in Ocular Tissues and Fluids (EVICT) study of survivors being evaluated for cataract surgery, 46 survivors with vision-impairing cataract tested negative for Ebola virus RNA in the aqueous humor at 19 months and 34 months after acute EVD (*18**,**19*). On the basis of those data, it is possible that EVD survivors have a greater risk for uveitis immediately after acute EVD as a result of viral persistence and that risk for uveitis decreases over time as the virus clears. Further studies will clarify the relationship between viremia, viral persistence in immune-privileged sites such as ocular tissues, and the development and progression of uveitis.

Nearly 20% of EVD survivors in this cohort had moderate vision impairment of 20/50 or worse in their better-seeing eye, which indicated ongoing ophthalmic disease at long-term follow-up. Ophthalmic findings associated with vision impairment in EVD survivors, including uveitis, cataract, corneal scar, and optic neuropathy, indicate a range of ocular disease that may contribute to visual illness and requires ongoing management. The EVICT study reported similar findings that vision-impairing cataract, posterior synechiae, optic neuropathy, and retinal detachment were strongly associated with worse logMAR VA in EVD survivors referred for vision impairment or cataract evaluation (*20*). In a population-based study conducted in 2021in Sierra Leone, 5.1% of participants were bilaterally blind and ≈16% had varying degrees of vision impairment (*21*). The most common causes of bilateral blindness in the general population of Sierra Leone were untreated cataracts (59.4%), glaucoma (21.7%), and nontrachomatous corneal opacity (8.4%). Of note, the participants in the population-based study were >50 years of age, whereas the median age in our cohort of EVD survivors was 29 years. In addition, uveitis was not reported as a major cause of blindness or vision impairment in the general population.

Cataracts were found in 11.2% of eyes in our cohort and were strongly associated with vision impairment. Our analysis showed that uveitis found in the study exam was associated with a previous diagnosis of cataract (p<0.001); ≈10% of newly diagnosed cataracts were uveitic in nature. Considering the relatively young age of this cohort, a substantial number of the cataracts are likely attributable to previous or ongoing inflammation or treatment with topical corticosteroids. Cataract development related to uveitis was reported in other studies. Tiffany et al. (*12*) showed that 7 out of 8 EVD survivors with cataracts had concurrent uveitis. Mattia et al. (*11*) found that 10% of EVD survivors with uveitis had concurrent early cataracts and were relatively young (median age 29 years), suggesting that that the cataracts were not related to age. Thus, early detection and treatment for uveitis in EVD survivors could reduce the frequency of cataract development and improve visual outcomes.

Corneal scar and optic neuropathy were less common in EVD survivors but were also strongly associated with vision impairment status. A post hoc analysis of the EVICT study corroborated findings of worse logMAR VA in survivors with optic nerve disease than those without optic nerve disease (*22*). However, the presence of ocular surface disease, including dry eye, band keratopathy, and corneal scar, were not strongly associated with worse visual outcomes (*23*). Further studies will determine whether these ophthalmic manifestations are more common in EVD survivors than control participants and are associated with worse vision. To understand the prevalence of uveitis, visual impairment, and disease pathogenesis of ocular inflammation in EVD survivors compared with close-contact control patients, a longer-term study is currently underway in Sierra Leone (*24*).

Limitations of our study include the retrospective study design and lack of a control group. Timing or directionality of associations cannot be determined because this was a cross-sectional study. In addition, Ebola serum IgG was not available, so EVD survivor status was determined by survivor certificates and a history of ETU admission. Finally, there is also a potential for selection bias for symptomatic survivors that could skew the results toward a higher prevalence of ophthalmic findings; however, we recruited for examination any EVD survivor in the targeted regions who could be reached.

In summary, this study identified a range of ophthalmic complications that were associated with vision impairment in EVD survivors >3 years after the resolution of their acute illness. Uveitis remained one of the most common ophthalmic findings in EVD survivors, but cataract, corneal scars, and optic neuropathy were also found to be associated with poor visual outcomes. Our findings highlight the need for long-term vision care and follow-up for uveitis as well as a range of ophthalmic conditions in EVD survivors to optimize their visual potential and associated quality of life.
